# Printed Flexible Plastic Microchip for Viral Load Measurement through Quantitative Detection of Viruses in Plasma and Saliva

**DOI:** 10.1038/srep09919

**Published:** 2015-06-05

**Authors:** Hadi Shafiee, Manoj Kumar Kanakasabapathy, Franceline Juillard, Mert Keser, Magesh Sadasivam, Mehmet Yuksekkaya, Emily Hanhauser, Timothy J. Henrich, Daniel R. Kuritzkes, Kenneth M. Kaye, Utkan Demirci

**Affiliations:** 1 Division of Biomedical Engineering, Division of Renal Medicine, Department of Medicine, Brigham and Women’s Hospital, Harvard Medical School, Boston, MA, USA; 2 Harvard-MIT Division of Health Sciences and Technology, Cambridge, MA, USA; 3 Department of Medicine, Brigham and Women’s Hospital, Harvard Medical School, Boston, MA, USA; 4 Division of Infectious Diseases, Brigham and Women’s Hospital, Harvard Medical School, MA, USA; 5 Department of Radiology, Canary Center at Stanford for Cancer Early Detection, Stanford University School of Medicine, Palo Alto, CA, USA

## Abstract

We report a biosensing platform for viral load measurement through electrical sensing of viruses on a flexible plastic microchip with printed electrodes. Point-of-care (POC) viral load measurement is of paramount importance with significant impact on a broad range of applications, including infectious disease diagnostics and treatment monitoring specifically in resource-constrained settings. Here, we present a broadly applicable and inexpensive biosensing technology for accurate quantification of bioagents, including viruses in biological samples, such as plasma and artificial saliva, at clinically relevant concentrations. Our microchip fabrication is simple and mass-producible as we print microelectrodes on flexible plastic substrates using conductive inks. We evaluated the microchip technology by detecting and quantifying multiple Human Immunodeficiency Virus (HIV) subtypes (A, B, C, D, E, G, and panel), Epstein-Barr Virus (EBV), and Kaposi’s Sarcoma-associated Herpes Virus (KSHV) in a fingerprick volume (50 µL) of PBS, plasma, and artificial saliva samples for a broad range of virus concentrations between 10^2^ copies/mL and 10^7^ copies/mL. We have also evaluated the microchip platform with discarded, de-identified HIV-infected patient samples by comparing our microchip viral load measurement results with reverse transcriptase-quantitative polymerase chain reaction (RT-qPCR) as the gold standard method using Bland-Altman Analysis.

The development of sensitive, portable, rapid, and cost-effective virus detection and quantification in biological samples has broad diagnostic and biosafety applications in food safety, homeland security, and infectious disease diagnosis^1^,^2^,^3^. It has the potential to revolutionize personal healthcare in resource-constrained settings as well as developed countries. The development of such point-of-care (POC) assays for viral load measurement is currently a significant technical and clinical challenge and requires innovative and urgent solutions at the interface of engineering and medicine^4^,^5^. Commercially available assays for sensitive viral load measurement, including reverse transcriptase-quantitative polymerase chain reaction (RT-qPCR), require nucleic acid amplification or signal amplification, which are labor-intensive, expensive, time-consuming, and complex. Despite the recent advances in micro- and nano-technologies, which have shown a great promise for the development of POC diagnostic platforms, there is currently no commercially available biosensor for POC viral load measurement that is rapid, sensitive, inexpensive, and easy-to-use suitable for resource-constrained settings^6^,^7^,^8^,^9^,^10^,^11^,^12^. A major challenge in developing POC viral load measurement platforms is the sensitive and specific detection and quantification of viruses using a fingerprick volume (<100 µL) of biological samples at clinically relevant concentrations without the need for amplification and sample preprocessing. Here, we present a printed flexible plastic microchip technology that can capture and quantify viruses in a variety of biological samples, including plasma and artificial saliva, through electrical sensing of viral lysate. The integration of electrical sensing with printed flexible plastic microfluidics can potentially provide a promising opportunity to develop an innovative viral load measurement platform that addresses the current clinical challenges associated with viral load testing^1^,^14^,^15^,^16^,^17^,^18^,^19^,^20^. To demonstrate the broad applications of this biosensing platform, we evaluated our microchip technology to detect and quantify multiple virus targets, including Human Immunodeficiency Virus-1 (HIV-1) (subtypes A, B, C, D, E, G, and panel), Epstein-Barr Virus (EBV), and Kaposi’s Sarcoma-associated Herpes Virus (KSHV) in spiked plasma and artificial saliva samples as well as HIV-infected patient samples.

## Results

### Viral load measurement – HIV

**Figure 1:**
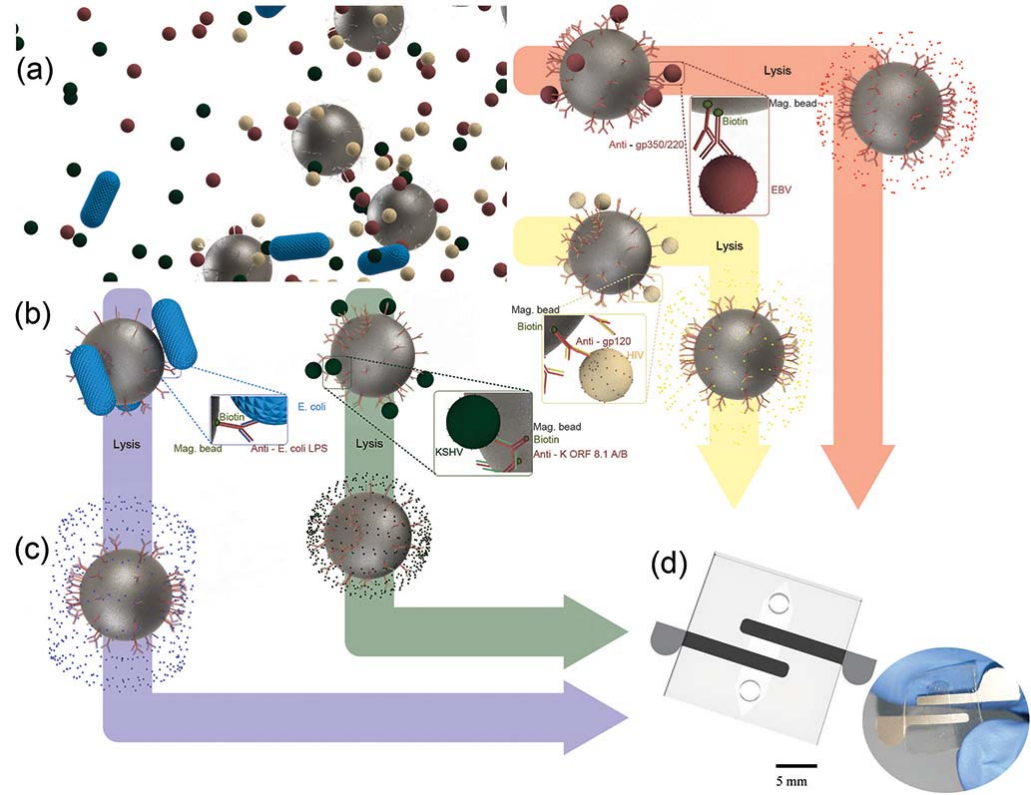
3D Schematic of pathogen capture and detection on a printed flexible plastic microchip. **(a)** Magnetic beads conjugated with antibodies to capture HIV, EBV, KSHV or *E. coli* are mixed with a fingerprick volume of a biological sample off-chip. **(b)** Captured pathogens are washed 4 times with a low conducting media (glycerol) to remove residual high electrically conductive background. **(c)** The captured bioagents are then lysed with Triton x-100 **(d)** The pathogen lysate is then injected into the chip for capacitance measurement. The captured and isolated pathogens are detected through capacitance spectroscopy of the lysate on a printed flexible plastic microchip.

**Figure 2:**
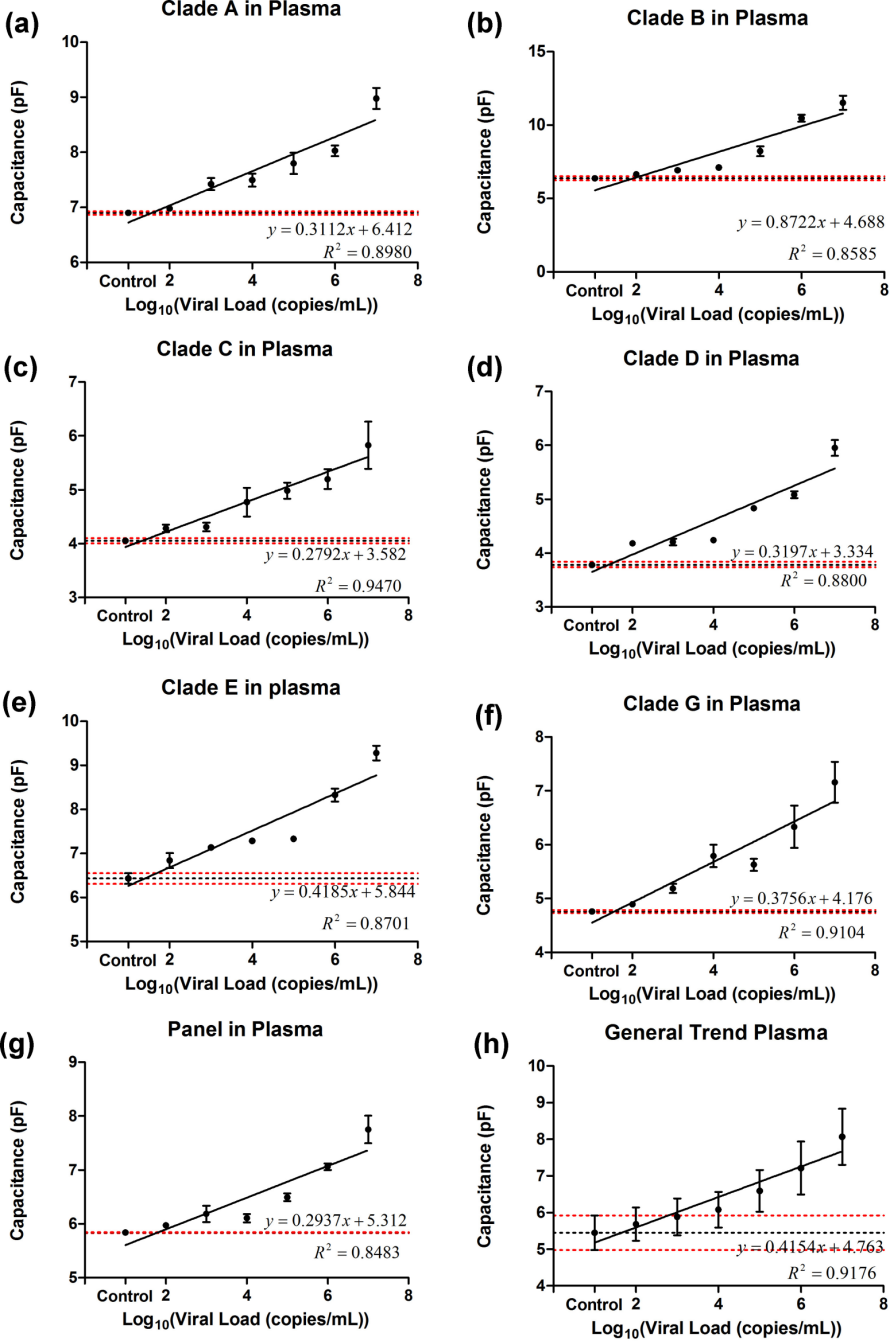
Microchip evaluation using HIV-1 subtypes spiked in plasma. Linear correlation between viral load of the samples and on-chip capacitance measurements at 2 V and 1 KHz for multiple HIV-1 subtypes A **(a)**, B **(b)**, C **(c)**, D **(d)**, E **(e)**, G **(f)**, and panel **(g)** showed coefficient of determinations between 0.83 and 0.94. The lowest dilutions that showed significant capacitance change compared to virus-free control plasma samples were 10^3^ (p = 0.001, n = 8), 10^3^ (p = 0.008, n = 8), 10^2^ (p = 0.049, n = 8), 10^2^ (p = 0.0002, n = 8), 10^3^ (p = 0.002, n = 8), 10^3^ (p = 0.0007, n = 8) and 10^4^ (p = 0.004, n = 8) for viral load measurement in plasma samples spiked with HIV-1 subtypes A **(a)**, B **(b)**, C **(c)**, D **(d)**, E **(e)**, G **(f)**, and panel **(g)**, respectively. The limits of detection were established as the lowest concentration from the experimental results with a statistically significant difference (p<0.05) from virus-free control sample calculated through Mann-Whitney method of analysis. **(h)** The general trend of all subtypes of HIV-spiked plasma samples was obtained by generating a simple linear regression using all of the experimental results obtained from HIV-1 subtypes A, B, C, D, E, G, and panel. Error bars represent standard error of mean. Control samples were pathogen-free plasma samples. Horizontal black and red dotted lines represent the average capacitance magnitude of the control samples and control ± standard error, respectively.

Figure 1 shows the 3D schematics of the presented technology for bioagent capture and detection on a printed flexible plastic microchip through capacitance spectroscopy of bioagent lysate. To evaluate the ability of the microchip to detect a broad range of targets in biological samples with clinically relevant concentrations, we first demonstrated HIV viral load measurement in a fingerprick volume (50 µL) of spiked-Phosphate Buffered Saline (PBS) and plasma samples for multiple HIV-1 subtypes (A, B, C, D, E, G, and panel) (Fig. 2). HIV-1 particles were captured by biotinylated polyclonal anti-gp120 antibodies anchored to streptavidin-coated magnetic beads (Fig. 1). Captured viruses were washed with a low-electrically conductive solution (10% glycerol), and lysed using 1% Triton x-100. The virus lysis step releases the charged molecules into the lysis buffer, *i.e.* Triton x-100, which changes the bulk electrical properties of the solution. Captured viruses were detected through capacitance spectroscopy of viral lysate samples on a flexible plastic microchip with screen-printed electrodes. Our preliminary experimental results, using diluted PBS in deionized (DI) water samples, demonstrated that the impedance magnitude of the samples decreases, while the capacitance magnitude increases due to an increase in electrical conductivity of the samples (Supplementary Fig. S1a, b). These results also demonstrated that capacitance spectroscopy in our microchips provided a more sensitive detection method as compared to impedance spectroscopy at 1,000 Hz (Supplementary Fig. S1c). Moreover, these results show that the maximum capacitance magnitude change in the samples occurred at frequencies between 100 Hz and 1,000 Hz (**Supplementary Fig. S2**). We chose 1,000 Hz as the detection frequency for viral load measurements as we observed lower noise at this frequency. We measured the capacitance magnitude response due to virus lysis at 2V and 1,000 Hz in HIV-spiked PBS (**Supplementary Fig. S3**) and HIV-spiked plasma (Fig. 2) samples with clinically relevant virus dilutions between 10^2^ copies/mL and 10^7^ copies/mL. The lowest diluted virus concentrations in HIV-spiked PBS samples with statistically significant capacitance change compared to the control samples were 10^3^ copies/mL (p = 0.0005, n = 8), 10^2^ copies/mL (p = 0.0104, n = 8), 10^3^ copies/mL (p = 0.0007, n = 8), 10^3^ copies/mL (p = 0.0002, n = 8), 10^2^ copies/mL (p = 0.007, n = 8), 10^3^ copies/mL (p = 0.002, n = 8), and 10^3^ copies/mL (p = 0.010, n = 8) for subtypes A, B, C, D, E, G, and panel, respectively (Fig. S3).

We also observed that the lowest diluted virus concentrations in HIV-spiked plasma samples with statistically significant capacitance change compared to the control samples were 10^3^ copies/mL (p = 0.001, n = 8), 10^3^ copies/mL (p = 0.008, n = 8), 10^2^ copies/mL (p = 0.049, n = 8), 10^2^ copies/mL (p = 0.0002, n = 8), 10^3^ copies/mL (p = 0.002, n = 8), 10^3^ copies/mL (p = 0.0007, n = 8), 10^4^ copies/mL (p = 0.004, n = 8) for HIV-1 subtypes A, B, C, D, E, G, and panel, respectively (Fig. 2). The different detection limits for HIV subtypes may be due to the differences between the HIV-1 gp120 envelope sequences of the subtypes used in this study as compared to the sequence of the immobilized anti-gp120 antibody used for virus capture. To demonstrate this difference, we report the gp120 sequence of the HIV subtypes A, B, D, E, and G aligned with the EU541617 reference strain used to generate the polyclonal anti-gp120 antibody (Supplementary Fig. S4). The inherent cumulative inaccuracy of serial dilution can affect the coefficients of determination of our samples making limit of detection determination challenging.

### Viral load measurement - EBV and KSHV

**Figure 3:**
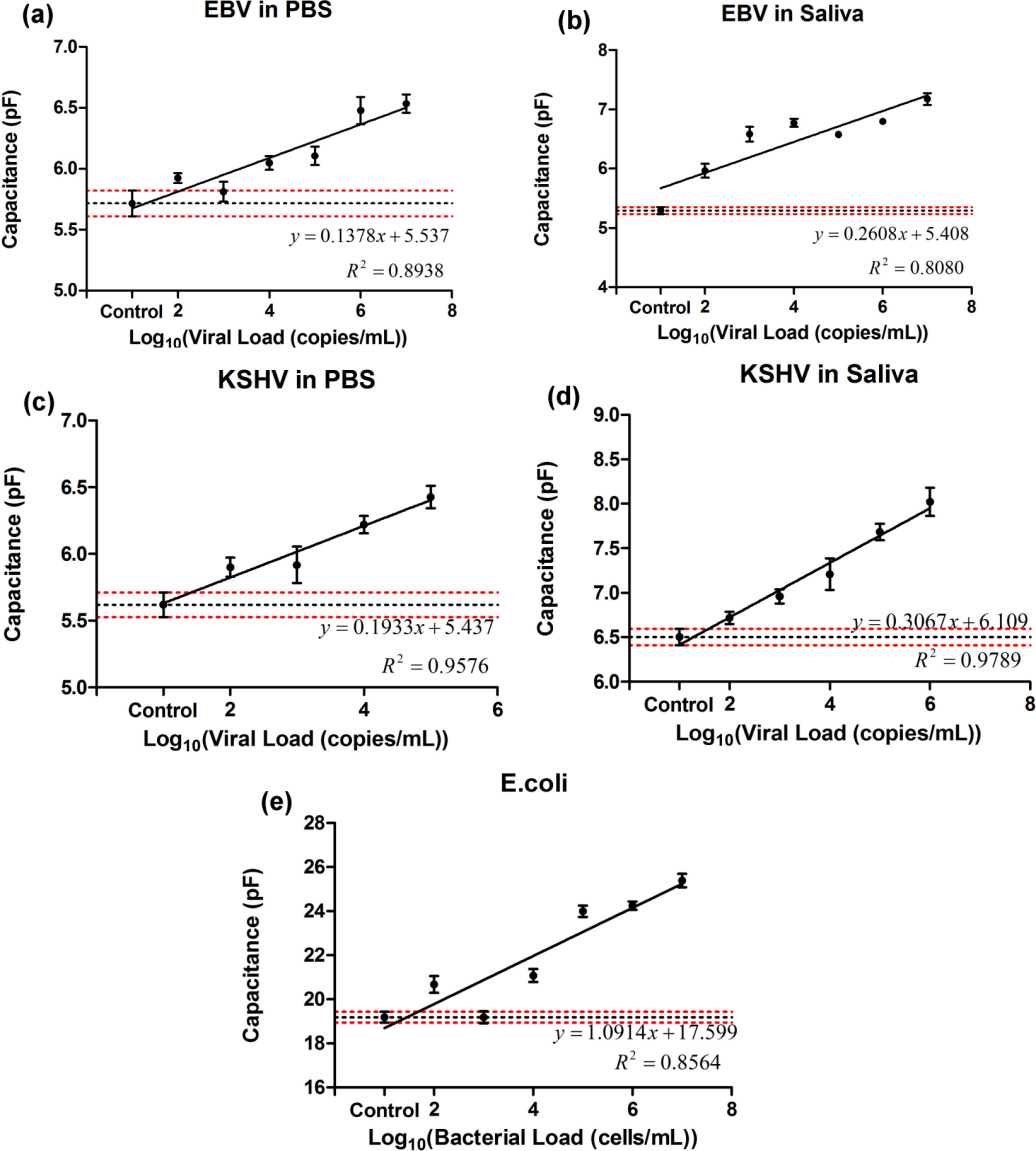
Microchip evaluation using EBV and KSHV spiked in PBS and saliva and *E.coli* spiked in saliva samples. Linear correlations between viral load of samples and on-chip capacitance measurements at 2 V and 1 KHz for EBV-spiked PBS **(a)** and saliva **(b)** samples were calculated. The lowest dilutions with significant capacitance change as compared to virus-free control samples were 10^4^ copies/mL in EBV-spiked PBS (**a**, p = 0.0070, n = 8) and 10^2^ copies/mL in **EBV-spiked** saliva (**b**, p = 0.0007, n = 8) samples. Linear correlations between viral load of samples and on-chip capacitance measurements at 2 V and 1 KHz for KSHV-spiked PBS **(c)** and saliva **(d)** samples were calculated. The lowest dilutions with significant capacitance change as compared to virus-free control samples were 10^2^ copies/mL (p = 0.0426, n = 8) and 10^3^ copies/mL (p = 0.0047, n = 8) for KSHV-spiked PBS and saliva samples, respectively. Error bars represent standard error of mean. **(e)** *E. coli* was spiked in artificial saliva. 5% Triton was used in contrast to the 1% Triton used for viral lysis. The lowest statistically significant dilution of stock is 10^4^ (p = 0.0013, n = 8) and was calculated utilizing Mann-Whitney analysis. Error bars represent SEM. Control samples were pathogen-free **(a, c)** PBS and **(b, d, e)** artificial saliva samples. Horizontal black and red dotted lines represent the average capacitance magnitude of the control samples and control ± standard error, respectively.

To demonstrate the broad applicability of the microchip technology, we also evaluated the platform to detect and quantify EBV and KSHV spiked in PBS and artificial saliva samples. EBV and KSHV particles were selectively captured on streptavidin-coated magnetic beads conjugated with biotinylated MA-gp350/250 and MAb-K ORF 8.1A/B antibodies, respectively. The linear correlation between the capacitance magnitude of the viral lysate and viral load of PBS and artificial saliva samples spiked with EBV and KSHV are reported in Figure 3. The samples were spiked with viruses at concentrations ranging from 10^2^ copies/mL to 10^7^ copies/mL. The lowest diluted virus concentrations in EBV-spiked PBS and artificial saliva samples with statistically significant capacitance change compared to the control samples were 10^4^ copies/mL (p = 0.0070, n = 8) and 10^2^ copies/mL (p = 0.0007, n = 8), respectively (Fig. 3a, b). We also observed that the lowest diluted virus concentrations in KSHV-spiked PBS and artificial saliva samples with statistically significant capacitance change compared to the control samples were 10^2^ copies/mL (p = 0.0426, n = 8) and 10^3^ copies/mL (p = 0.0047, n = 8), respectively (Fig. 3c, d). Therefore, the presented microchip technology satisfies the clinical requirement to detect EBV and KSHV^21^,^22^.

### Detection of bacterial pathogens

This microchip technology has the potential to selectively detect pathogens other than viruses, including bacterial pathogens. Here, we present our results on capture and detection of *E. coli* in artificial saliva using the printed flexible plastic microchips. *E. coli* cells were selectively captured on magnetic beads conjugated with anti-*E. coli* LPS antibody in 50 µL of diluted artificial saliva samples. The lowest diluted cell concentration in *E. coli*-spiked artificial saliva samples with statistically significant capacitance change compared to the control samples was 10^4^ cells/mL (p = 0.0013, n = 8) (Fig. 3e).

### Specificity evaluation

**Figure 4:**
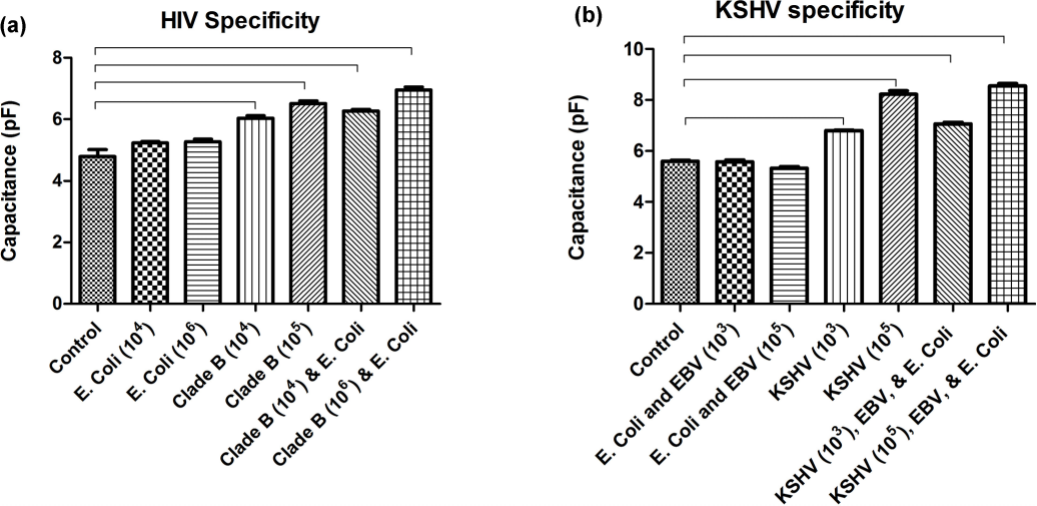
Specificity evaluations of the microchip. **(a)** HIV-1 subtype B was specifically captured and detected in the presence of *E. coli* spiked in plasma samples. *E. coli* at concentrations of 10^4^ cells/mL and 10^6^ cells/mL showed no significant difference (p>0.05, n = 4) against HIV-free control. HIV-spiked samples (10^4^ copies/mL and 10^5^ copies/mL) and the mixture of HIV-1 and *E. coli* samples showed statistically different (p<0.05, n = 4) capacitance values compared to virus-free control samples. **(b)** KSHV was specifically captured and isolated from a mixture of KSHV, EBV and *E. coli* in artificial saliva. Mixtures of EBV (10^3^ copies/mL and 10^5^ copies/mL) and *E. coli* (10^3^ cells/mL and 10^5^ cells/mL) showed no significant difference (p>0.05, n = 4) in capacitance magnitude from KSHV-free control samples. KSHV-spiked samples at concentrations of 10^3^ copies/mL and 10^5^ copies/mL showed a significant difference (p<0.05, n = 4) from control. The capacitance magnitude of the mixture of KSHV, EBV, and *E. coli* samples were also significantly different than control samples. Brackets indicate statistically significant capacitance magnitude shift between the linked groups. Control samples were pathogen-free **(a)** plasma and **(b)** artificial saliva samples. Error bars represent standard error of mean (n = 4).

To evaluate the specificity of the presented microchip technology, we tested the platform to detect a target virus such as HIV or KSHV in the presence of other bioagents such as EBV and *E. coli*. We have previously shown that HIV can be specifically detected in the presence of other viruses, including EBV in samples using electrical sensing of viral lysate on-chip^9^. Here, we also studied if the presence of non-target pathogens such as *E. coli*, which are much larger in size, may release a significantly higher amount of ions into the solution. Control samples in our specificity evaluation experiments were pathogen-free plasma and artificial saliva samples. Figure 4a shows the specificity evaluation results for HIV detection in plasma samples in the presence of *E. coli.* In this experiment, we used magnetic beads conjugated with anti-gp120 antibody to selectively capture HIV. The capacitance magnitude of *E. coli* samples without HIV was not statistically different than control samples (p>0.05, n = 4) with a statistical power of 0.68. However, the capacitance magnitude of the mixture of HIV subtype B and *E. coli* was significantly different than control and *E. coli* samples (p<0.05, n = 4) (Fig. 4a). These results showed that anti-gp120 antibody specifically captured and isolated HIV and not *E. coli* in the samples. Figure 4b shows the specificity evaluation of the microchip for KSHV detection in the presence of EBV and *E. coli*. We used anti-ORF K 8.1 A/B antibody for microchip specificity evaluation to detect KSHV. The capacitance magnitude of the mixture of *E. coli* and EBV did not show a statistically significant difference (p>0.05, n = 4) compared to the control samples with a statistical power of 0.73, whereas the capacitance magnitude of the mixture of KSHV, EBV, and *E. coli* was significantly different than control samples, as well as the mixture of EBV and *E. coli* samples (p<0.05, n = 4) (Fig. 4b). This is due to highly specific capture and isolation of KSHV in the samples using anti-ORF K 8.1 A/B antibody.

### Patient samples

**Figure 5:**
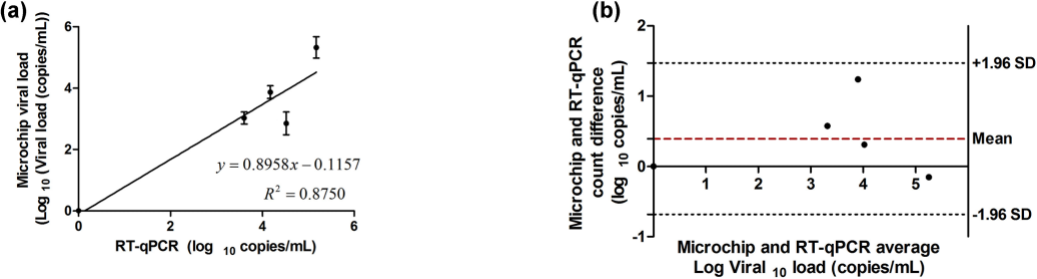
Device validation with patient samples. **(a)** Correlation data of microchip viral load and the viral load obtained through RT-qPCR. Viral load of zero was considered for patients with undetectable loads. Error bars represent standard error of mean (n = 8). **(b)** Bland-Altman comparison of viral loads obtained through RT-qPCR and our printed flexible plastic microchip. Bland-Altman analysis did not show any evidence of systematic bias. Quantitative information is available in supplementary Table S2.

We also evaluated our viral load measurement using five anonymous, discarded HIV-infected patient plasma samples. We first generated a standard curve by performing a least-square fit linear regression for HIV viral load measurement using the experimental results with the HIV-spiked samples reported in **Supplementary Figures S3a-g** (**Fig. S3h**). We used this standard curve to translate the capacitance magnitude measurement to viral load of the patient samples tested in this study. The quantitative HIV viral load measurement results for the five patient samples using our microchip platform and RT-qPCR are presented in **Supplementary Table S2**. The capacitance magnitude change for one of the patient samples, which had undetectable viral load by means of RT-qPCR, was statistically negligible as compared to the control samples in our microchip. The data point related to the viral load of this patient sample is shown at the origin of Figure 5a. These results were analyzed and statistically evaluated using the Bland-Altman method^23^,^24^ and presented in Figure 5b. These results confirmed that there was no evidence for a systematic bias for viral load measurement in HIV-infected patient samples using our printed flexible plastic microchip technology.

## Discussion

We have previously shown that impedance magnitude of viral lysate can be used for virus detection on microfluidic devices with gold microelectrodes fabricated on glass substrates using lift-off lithography method^9^. We detected HIV-1 spiked in PBS samples with virus concentrations on the order of 10^8^ copies/mL. Here, we demonstrate a new microchip technology for virus detection and quantification by integrating screen-printing silver microelectrodes on flexible plastic substrates and capacitance spectroscopy. Double layer capacitance at low frequencies, such as 1,000 Hz, in electrically low conductive solutions, such as the viral lysate samples used in this work, changes more significantly as the ion concentration in the sample increases compared to impedance magnitude^25^. We have evaluated our technology using multiple bioagents, including HIV, EBV, KSHV, and *E. coli*. Our results show that this microchip technology can detect viruses in complex biological samples, such as human plasma and artificial saliva, with clinically relevant virus concentrations. This microchip technology can be used for acute HIV detection, when maximum viral replication occurs^26^, as well as for treatment failure diagnosis based on POC ART monitoring defined by the World Health Organziation (WHO)^13^. Further technology development is required to enhance the sensitivity comparable with PCR-based assays. This biosensing platform can be potentially used for detection and quantification of other viral particles that have well-described biomarkers and antibodies for capture, including herpes, influenza, malaria, and tuberculosis.

The presented microchip technology is a platform technology to detect and quantify multiple viruses such as HIV, EBV, and KSHV. It can potentially detect multiple viruses at the same time and on a single chip with parallel microchannels. In such a device, each microchannel has a separate pair of microelectrodes for electrical measurement and is functionalized with a specific antibody to capture a target virus for detection. Such microchip design enables multiplexing, however, the cost per test increases accordingly. Co-infections with other pathogens such as EBV, Cytomegalovirus (CMV), Human Papillomavirus (HPV), Herpes simplex virus (HSV), and Candida are also common in HIV-infected patients, specifically in those not receiving ART. Therefore, multiplexing on a single chip to detect these pathogens at the same time can have a significant impact on HIV management in developing countries. Future studies can evaluate the specificity of the electrical sensing microchip technology to detect HIV in the presence of CMV, HPV, HSV, and Candida in plasma.

To prepare this microchip technology for field-testing, further technology development and optimization is required to perform all of the sample handling steps, including virus capture, washing, and lysis on-chip. It is also important to evaluate the effect of temperature, humidity, and stability of reactions at different sites on the performance of this microchip technology with respect to sensitivity, specificity, and reproducibility. Device throughput for viral load testing is also another design parameter that should be addressed during technology transfer and commercialization.

The electrical measurements in this study were performed using an impedance spectrometer to evaluate the performance of the microchip technology for AC frequencies between 100 Hz and 1 MHz. These results show that the electrical measurements can be done at a single frequency for multiple viruses, *i.e.* 1,000 Hz. Therefore, it is potentially possible to build a hand-held impedance/capacitance meter utilizing affordable miniaturized frequency oscillators, microcontrollers, and impedance/capacitance meter microchips available in the market.

Here, we evaluated our printed flexible plastic microchip with respect to sensitivity, specificity, and linearity. To validate this technology, other parameters, including repeatability, robustness, precision, system suitability, and performance over time should also be evaluated. Furthermore, it is also required to evaluate the technology with other HIV subtypes, including the less common members of group M (K, J, F, H, I), N, and O, as well as HIV-2 (groups A and B).

Viral load measurement is critical in infectious disease diagnosis and treatment monitoring, and can facilitate treatment expansion especially in resource-constrained settings. ART is a successful treatment to suppress HIV, prolong life in HIV-infected patients, and reduce HIV infection transmission rates, and has become even more affordable and accessible in developing countries^27^. However, a majority of HIV-infected individuals do not receive ART due to lack of affordable, rapid, and sensitive HIV diagnostic tools at the POC^28^. Virological suppression represents the most accurate immunological response of the patients to ART^29^. Thus, the most accurate and preferred method for ART monitoring to diagnose treatment failure, as recommended by the 2013 consolidated guidelines on the use of ART, is viral load measurement^30^. Viral load testing is important for identifying treatment failure and enabling medication changes or other interventions to prevent immunologic compromise and clinical progression of disease. POC viral load tests could be used to determine the efficacy of second line antiretroviral therapies in addition to identifying failure of initial regimens. Early detection of virological failure allows for both targeted adherence interventions and better preservation of the efficacy of second-line regimens. In addition, POC viral load testing may also be applied to the diagnosis of acute HIV infection and prompt initiation of ART. Prompt ART initiation may lead to a greater preservation of immune function and has the potential to reduce HIV-1 transmission. Currently available RNA- and PCR-based assays for viral load measurement in developed countries are expensive, laboratory-based, complex, time-consuming, and labor-intensive, and cannot be easily used in resource-constrained settings. p24 assays and CD4^+^ cell counting strategy cannot be used for monitoring the efficacy of treatment and detecting ART failure in patients^31^,^32^,^33^,^34^,^35^. Therefore, to detect early virological failure and to expand access to ART, new emerging technologies are urgently needed to facilitate viral load testing at the POC^36^,^37^,^38^,^39^,^40^. POC viral load testing may lead to increasing the adherence and minimizing the drug resistance through timely detection of virological failure^41^,^42^,^43^.

Kaposi’s Sarcoma-associated Herpes Virus (KSHV) and Epstein-Barr Virus (EBV) are human gamma herpesviruses^44^. KSHV and EBV are the causative agents of a number of malignancies, especially common in immunosuppressed individuals such as those with HIV/AIDS. KSHV is the etiologic agent of Kaposi’s sarcoma (KS)^45^,^46^,^47^ and primary effusion lymphoma (PEL)^48^,^49^, and is strongly associated with multicentric Castleman’s disease^50^,^51^, an aggressive lymphoproliferative disorder. KS is epidemic in sub Saharan Africa and is the leading AIDS malignancy^52^,^53^,^54^. EBV has a causative role in several human malignancies, including Burkitt's lymphoma^55^, Hodgkin's lymphoma^56^, nasopharyngeal carcinoma^57^, lymphoproliferative disease in immune-suppressed patients^58^, and lymphomas in HIV-infected patients^59^. There are no specific therapies available for these cancers, and therapy generally relies on cytotoxic chemotherapy or improvement of immune function. KSHV is frequently found in saliva of infected persons and oral contact is the most common route of transmission in endemic regions^60^,^61^,^62^. EBV also spreads through saliva and may initially replicate in the oropharyngeal epithelium^59^. However, the details of transmission are poorly understood. Rapid and efficient detection of KSHV and EBV in saliva would enable studies to better understand viral transmission. Such an understanding may lead to strategies to interrupt transmission of these viruses and prevent virus-associated malignancies. Detection of viral load in blood can also be useful to anticipate the onset of KSHV- and EBV-associated diseases or to monitor the response to therapies. In fact, elevated KSHV viral load in blood has been shown to predict the progression of KS^63^,^64^. In addition, several studies have shown a strong correlation between EBV burden in blood and presence of post-transplant lymphoproliferative disease (PTLD) in transplantation recipients^65^,^66^,^67^. Elevated EBV DNA in patients can be detected prior to PTLD diagnosis^68^.

Electrical sensing in microfluidics is insensitive to light intensity and does not need the bulky equipment usually required in optical sensing platforms, and is a powerful modality to create portable and sensitive biosensors^2^,^3^,^9^,^69^,^70^. Paper-based microfluidics has shown great promise in developing affordable, mass-producible, and disposable POC diagnostic assays for developing countries^14^,^71^. Paper substrates are light (~10 mg/cm^2^), thin, flexible, and disposable, and paper microchips can be fabricated for a few cents^14^,^72^,^73^,^74^. Here, we fabricated our microchips by screen-printing conductive silver ink on flexible hydrophobic plastic sheets that are thin, light, flexible, and inexpensive. These advantages are similar to those of paper-based microfluidics. In addition, our chip fabrication is less complicated as we do not use commonly used methods for paper-based microfluidic fabrication, including photolithography^19^, plotting^75^, inkjet etching^76^, plasma etching^77^, or wax printing^78^,^79^. We report a microchip technology that integrates electrical sensing and printed flexible plastic microfluidics for inexpensive and rapid viral load measurement through viral lysate capacitance spectroscopy. The material cost of the microchip include less than 1 cent for flexible plastic sheets, less than 10 cents for electrodes, and less than 1 cent for DSA. The material costs for magnetic beads and antibody (anti-gp120 antibody) are 80 cents and 87 cents per test, respectively. These costs do not include labor costs. Therefore, we present a new viral load testing technology with the material cost per microchip of less than $2. The current assay time in the laboratory can take up to an hour, including 30 minutes for sample incubation to capture viruses, 4 minutes for washing, 1 minute for virus lysis, 10 minutes for read-out and analysis, and transfer steps. In this microchip technology, viruses are captured and isolated from biological samples off-chip utilizing highly specific antibodies. The virus lysis step in this method releases charged molecules in viral particles into an electrically low conductive solution, *i.e.* Triton x-100. These charged molecules change the electrical properties of the bulk solution as well as double layer properties on the surface of the electrodes. Capacitance spectroscopy of the viral lysate solution on-chip reveals the virus concentration in the sample.

There are a variety of inhibitory factors with different concentrations in biological samples such as plasma, saliva, and semen. These inhibitory factors may have interference with the infectivity of the virus in body fluids. For instance, HIV can be reproduced and cultured in tissues and most of the body fluids such as plasma, breast milk, and genital secretions, except saliva^80^. Saliva is able to specifically suppress HIV-1^80^. However, saliva does not have a significant effect on the infectivity of other viruses such as EBV, Herpes simplex virus (HSV), and cytomegalovirus (CMV)^81^,^82^,^83^. Saliva is also the vehicle of person-to-person KSHV spread^60^,^84^,^85^. Inhibitory factors such as highly charged sulfated polysaccharides, secretory leukocyte protease inhibitor (SLPI), fibronectin, and thrombospondin 1 (TSP1) in whole saliva may interfere with the infectivity of HIV-1 through altering the CD4-gp120 interactions^80^,^86^. Such inhibitory factors may be also present in plasma, but in very low concentrations^80^,^87^. KSHV levels in blood can be quite variable, depending on the level of a patient's immunosuppression, and burden of disease^88^. Furthermore, inhibitory factors may interfere in enzyme-based assays or nucleic acid-based amplification methods. In our electrical sensing technology, we first capture and isolate intact viruses from artificial saliva or plasma using antibodies. The captured viruses are then washed several times to remove non-target bioagents, including inhibitory factors, before viral lysis and detection. The electrical sensing assay does not rely on enzyme-based processing or amplification of nucleic acids. We have not observed interference from such inhibitory factors in our studies using HIV-spiked plasma samples as well as HIV-infected patient plasma samples. However, further evaluation is required to investigate the effect of inhibitory factors on the performance of the electrical sensing microchip technology. Viral load level in different body fluids such as plasma and saliva is dynamic and may or may not be correlated with each other^89^. For example, HIV load level in plasma and semen are correlated and can be influenced by multiple factors such as antiretroviral therapy and adherence, HIV treatment resistance, and the stage of HIV disease^89^. Also, HIV loads in plasma and saliva are poorly correlated^90^.

## Materials and Methods

### Reagents

Ultrapure grade (Type I) water was obtained through purification using Milli-Q® Academic A-10® (Millipore). Phosphate buffered saline (PBS) was obtained from Gibco (Grand Island, NY; 10010). Polyclonal biotinylated goat anti-gp120 (4.0 mg/mL) for HIV-1 capture was purchased from Abcam (Cambridge, MA; ab53937). Mouse monoclonal antibodies reactive to proteins encoded by ORF K 8.1 A/B (0.86 mg/mL) of HHV-8 was purchased from Advanced biotechnologies (Columbia, MD; 13-212-100). Mouse monoclonal antibodies reactive to 350/220 KDa viral glycoprotein of EBV were purchased from Millipore (Billerica, MA; MAB10219). Mouse monoclonal antibodies reactive to *E. coli* lipopolysaccharide (0.5 mg/mL) for *E. coli* capture were acquired from Abcam (Cambridge, MA; AB35654). Glycerol (56-81-5) and Triton x-100 (9002-93-1) were both purchased from Sigma (St. Louis, MO). RPMI-1640 with L-Glutamine (10-040) was purchased from Corning (Manassas, VA). Fetal bovine serum (100-106) was obtained from Gemini, penicillin/streptomycin (15070-063) was purchased from Invitrogen, HEPES buffer (15630-080) was purchased from Life Technologies (*Grand Island, NY*), and recombinant human interleukin-2 (11011456001) was purchased from Roche.

### Preparation of R20/IL-2

R20/IL-2 contains RPMI-1640 with L-Glutamine (300 mg/mL), penicillin/streptomycin (50 U/mL 50 μg/mL), fetal bovine serum (heat inactivated) 20%, 10 mM of HEPES buffer, and 100 U/mL of recombinant human interleukin-2.

### Artificial saliva preparation

Artificial saliva prepared for these experiments has the following composition: 0.1 L each of 25 mM K_2_HPO_4_ (Sigma, 7758-11-4), 24 mM Na_2_HPO_4_ (Sigma, 7558-79-4), 150 mM KHCO_3_ (Sigma, 298-14-6), 1.5 mM MgCl_2_ (Sigma, 7786-30-3), 0.1 L of 100 mM NaCl (Sigma, 7647-14-5), 0.006 L of 25 mM citric acid (Sigma, 77-92-9), and 0.1 L of 5 mM CaCl_2_ (Sigma, 10043-52-4). The pH of the solution was maintained at 6.7 through the addition of NaOH (Sigma-Aldrich, 1310-73-2) or HCl (Sigma, 7647-01-0). Although this artificial saliva solution does not have proteins and enzymes, which are present in saliva making it a complex fluid, the artificial saliva prepared here is a model system that mimics ionic strength and ionic contents in saliva.

### HIV-spiked plasma samples preparation

Human whole blood from HIV-free donors was acquired from Research Blood Components, LLC, Cambridge, MA. Plasma was separated from whole blood samples through centrifugation at 1,500 rpm for 15 minutes. Plasma samples were spiked with viruses at stock concentration of 10^8^ copies/mL and serially diluted to prepare samples with diluted virus concentrations between 10^2^ copies/mL and 10^7^ copies/mL.

### HIV-infected patient samples

Discarded, de-identified clinical research plasma specimens from HIV-infected patients were used in this study. Informed consent was obtained from all patients prior to sample collection. Blood was collected by peripheral venipuncture. After initial centrifugation at 1,500 rpm for 15 minutes to separate the cellular and plasma components, the plasma was removed and centrifuged an additional time to remove possible debris. The resulting plasma was frozen and stored at −80°C until use. Patient samples (1 mL) were sent to the Infectious Diseases Clinic at the Brigham and Women’s Hospital for viral load measurement using Roche-COBAS AmpliPrep/COBAS TaqMan HIV-1 test, v2.0. The study protocol involving human subjects was approved by the Institutional Review Board of the Brigham and Women’s Hospital, Harvard Medical School. All experiments involving human subjects were performed in accordance with the relevant approved guidelines and regulations.

### Printed flexible plastic microchip fabrication

Transparency sheets (3M, St. Paul, Minnesota, CG5000) were used as the substrates of the microchip. With the use of a laser cutter (Universal Laser systems Inc., VLS 2.3, Scottsdale, AZ), inlet and outlets were cut into transparency sheets with a radius of 0.65 mm^91^. The power and scan rate at a height of 3.175 mm were at 83 W and 6 mm/s, respectively. The channel (12 mm × 5 mm) was cut into a double-sided adhesive (DSA; 3M; 8213) of 80 µm thickness. The power and scan speed were 10 W and 6 mm/s respectively, with the laser at a height of 3.175 mm. Silicone adhesive (Loctite, Rocky Hill, CT; 341-882) and silver-vinyl ink (<0.015 Ω/sq/mil; ECM, Delaware, OH; CI-1001) pastes were mixed in a ratio of 1:5 (w/w) to make silver paste. The dimensions of the electrodes (2 cm × 2 mm and separated by 2 mm) were cut into the masking paper (Mask-ease, Blick Art Materials; 44908-1003) with the laser cutter. The masking paper was taped over the transparency sheet and the silver paste mixture was applied and was smeared using a glass slide to create a uniform layer of the ink on top of the paper substrate (Supplementary Fig. S5). The masking tape was removed and the sheet was baked in an oven at 175°C for 3 hours. The transparency sheet with the printed electrodes was used as a base substrate of the microchip. The DSA layer, with the microchannel designs cut on it, was then attached to the paper substrate with printed electrodes and another transparency paper was attached to the other side.

### Magnetic bead preparation

200 µL of streptavidin-coated magnetic beads (Thermo Scientific, Rockford, IL; 88817) of 1 µm diameter was washed three times with PBS in a microcentrifuge tube. A Magna GrIP™ (Millipore) magnetic stand was used to isolate magnetic beads during washing. 10 µL of biotinylated target antibody was added to the solution. The mixture was incubated over-night on a shaker at 4°C.

### Target bio-agent capture with conjugated beads

The antibody conjugated magnetic beads were washed twice with PBS and resuspended in 2 mL PBS to remove any unconjugated antibody in the solution. 50 µL of magnetic beads was taken in a microcentrifuge tube. The supernatant was removed with the help of Magna GrIP™ and was replaced with 50 µL of the pathogen-spiked PBS, plasma, and plasma sample. This sample-bead mixture was incubated at room temperature for half an hour on a rotator (15 rpm). Control samples for the experiments were prepared by mixing 50 µL of virus-free PBS/saliva/plasma with magnetic beads.

### Washing and viral lysis

The sample-bead mixture was washed with 10% (v/v) glycerol 4 times off-chip following the room temperature incubation. The washing step effectively removes electrically conductive solution from the sample. This is critical in reducing sources of noise in the capacitance measurement results. The captured viruses were then lysed off-chip by replacing the glycerol with 50 µL of 1% (v/v) Triton X-100, which helps in breaking down the lipid bilayer. The sample was incubated at room temperature with 1% Triton X-100 for 1 minute prior to capacitance measurement on-chip.

### Capacitance measurement

20 µL of viral lysate was injected into the printed flexible plastic microchip and capacitance was measured using an LCR meter (LCR8110G, GWInstek, CA) for frequencies between 100 Hz and 1 MHz and 2 V.

### HIV culture

NIH provided all HIV-1 subtypes (A, B, C, D, E and G) and panel (circulating recombinant forms, CRF01_AE and CRF02_AG) under the research and reagent program. All subtypes were isolated from both Uganda and the United States. The panel of isolates contains six strains of HIV-1 that are globally prevalent. Standard co-culture methods were used to prepare viral stocks. Peripheral blood mononuclear cells (PBMC) were extracted from donors (HIV-1 negative) using the Ficoll Hypaque technique. PBMCs extracted from both HIV negative and HIV positive donors were co-cultured after stimulation with PHA (0.25 µg/mL) for 3 days. 100 U/mL of R20/IL-2 was used to maintain cultures incubated at 37°C with 5% CO_2_ atmosphere. Culture supernatants were replaced by fresh medium once every week. p24 levels of the supernatant were tested and when they reached 20 ng/mL, cultures were terminated. Viral stocks were serially diluted in PBS and plasma to prepare spiked samples with virus concentrations ranging from 10^2^ copies/mL to 10^7^ copies/mL. Roche-COBAS AmpliPrep/COBAS TaqMan HIV-1 test, v2.0 was used for viral load measurement in the stock virus samples at the Brigham and Women’s Hospital.

### KSHV and EBV production

iSLK.219^92^ cells (Gift from Don Ganem) were cultured in DMEM medium supplemented with 10% bovine growth serum (BGS) (HyClone) and 15 μg/ml gentamicin. iSLK.219 cells were cultured in the presence of 1 μg/mL puromycin and 250 μg/ml G418. B958 cells were cultured in RPMI medium supplemented with 10% bovine growth serum (BGS) (HyClone) and 15 μg/mL gentamicin.

Recombinant KSHV.BAC219 virus stocks were prepared from stable iSLK-219 cells induced by doxycycline (1 μg/mL) in the absence of puromycin, and G418. Three days later, supernatant was collected and cleared of cells and debris by filtration (0.45 μm). Virus particles were pelleted by centrifugation (13,300 g for 2 h at 4°C) using a Sorvall SLA-600TC rotor.

Infectious virus was assessed by titration of supernatants using infection of 293T cells. Cell-free virus supernatant was diluted in fresh medium (2 mL final per well) and used to inoculate 293T cells seeded at approximately 3.5 × 10^5^ cells/well in 6-well plates 24 h prior infection. Following inoculation, plates were immediately centrifuged (600 × *g* for 1h at 25°C), and supernatant was removed and replaced with fresh medium. The percentage of GFP-positive cells (from GFP-KSHV) was analyzed 48h later by fluorescence-activated cell sorting (FACS). Infectious units (IU) are expressed as the number of GFP-positive cells per mL of cell-free virus supernatant 48h after infection.

EBV virus stocks were prepared from B958^93^ induced by TPA (20 ng/mL) and sodium butyrate (32 mM). One day after induction, cells were centrifuged and media was replaced by fresh RPMI. Six days later, supernatant was collected and cleared of cells and debris by filtration (0.45 μm). Virus particles were collected by centrifugation (13,300 g for 2 h at 4°C) using a Sorvall SLA-600TC rotor.

### *E. coli* sample preparation and lysis conditions

*E. coli* was cultivated from the DH5α strain by incubating them overnight at 37°C in Luria-Bertani (LB) medium that is composed of 10 g/L of tryptone, 5 g/L of yeast extract and 10 g/L of NaCl. *E.coli* was separated by centrifugation at 10,000 g for 3 minutes and suspended in freshly prepared artificial saliva. *E.coli* lysis was performed with the same viral lysis protocol (see methods section) but with 5% Triton x-100 replacing 1% Triton x-100 as the lysing agent. Incubation after the addition of 5% Triton x-100 was at 60°C to improve the lysis of *E.coli*.

### Statistical analysis

The normality of the data collected for capacitance measurement was analyzed using Anderson-Darling test. Since the data is not normal, we selected non-parametric test for statistical analysis. Viral load comparisons between groups were analyzed using nonparametric Mann Whitney t test with threshold set at 0.05 (p< 0.05). Error bars represent standard error of the mean (SEM). We observed statistically significant difference with the sample sizes used in this study to evaluate the limit of detection (n = 4) of the microchip as well as microchip specificity (n = 8). A comparison study was performed as well with Bland-Altman analysis, between results from the printed flexible plastic microchips and results from RT-qPCR. Statistical analysis was performed using Prism (Version 5, Graphpad, La Jolla, CA).

## Supplementary Material

Supplementary Information Supplementary Information
