# Supplementary material for: Printed Flexible Plastic Microchip for Viral Load Measurement through Quantitative Detection of Viruses in Plasma and Saliva
Source: Sci Rep. 2015 Jun 5;5:9919. doi: 10.1038/srep09919 (PMC4456945; doi:10.1038/srep09919)
Supplement: Supplementary Information [file srep09919-s1.pdf]

Printed Flexible Plastic Microchip for Viral Load Measurement through Quantitative Detection of Viruses in Plasma and Saliva

Hadi Shafiee[1,2], Manoj Kumar Kanakasababapathy[1,2], Franceline Juillard[3], Mert Keser[1,2], Magesh Sadasivam[1,2], Mehmet Yuksekkaya[1,2], Emily Hanhauser[4], Timothy J. Henrich[4], Daniel R. Kuritzkes[4], Kenneth M. Kaye[3], Utkan Demirci[1, 5]*

[1] Division of Biomedical Engineering, Division of Renal Medicine, Department of Medicine, Brigham and Women's Hospital, Harvard Medical School, Boston, MA, USA.

[2] Harvard-MIT Division of Health Sciences and Technology, Cambridge, MA, USA.

[3] Department of Medicine, Brigham and Women's Hospital, Harvard Medical School, Boston, MA, USA

[4] Division of Infectious Diseases, Brigham and Women's Hospital, Harvard Medical School, MA, USA

[5] Department of Radiology, Canary Center at Stanford for Cancer Early Detection, Stanford University School of Medicine, Palo Alto, CA, USA

- Correspondence to: utkan@stanford.edu

To evaluate the impedance and capacitance magnitudes of samples with different electrical conductivities, we prepared diluted (v/v) PBS in DI water samples (0.01% PBS, 0.05% PBS, 0.1% PBS, and 0.5% PBS). We measured the impedance and capacitance magnitudes of these samples in the flexible plastic microchip. The results of these experiments are presented in Figures S1 and 2.

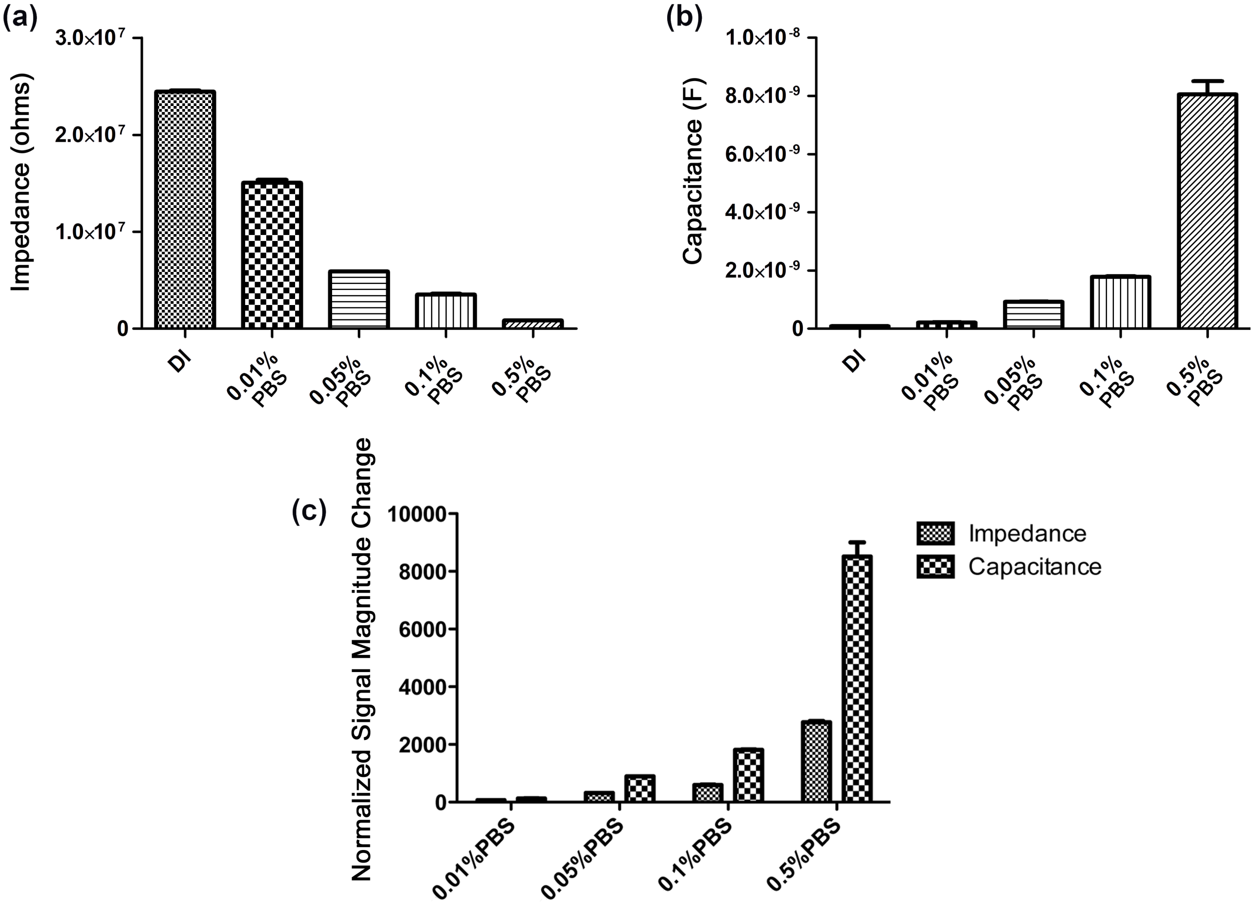

Figure S1. Comparison between Impedance and Capacitance measurements. Impedance **(a)** and capacitance **(b)** of various PBS concentrations (v/v dilutions in ultrapure grade (Type 1) water) were measured at 1 KHz and 2V on printed flexible plastic microchips. Impedance decreases with increasing concentrations while capacitance increases. **(c)** The normalized impedance magnitude change was measured by subtracting the impedance magnitude of the diluted PBS samples from the impedance magnitude of the DI sample divided by the impedance

magnitude of DI. The normalized capacitance magnitude change was measured by subtracting the capacitance magnitude of the diluted PBS samples from the capacitance magnitude of the DI sample divided by the capacitance magnitude of diluted sample. The signal magnitude changes were also multiplied by 100. The signal change between various concentrations of PBS and DI is a direct indication of the impedance magnitude change versus capacitance magnitude change on microchip sensitivity. The signal change for capacitance measurements is significantly greater than signal change due to impedance. Error bars represent SEM (n=3).

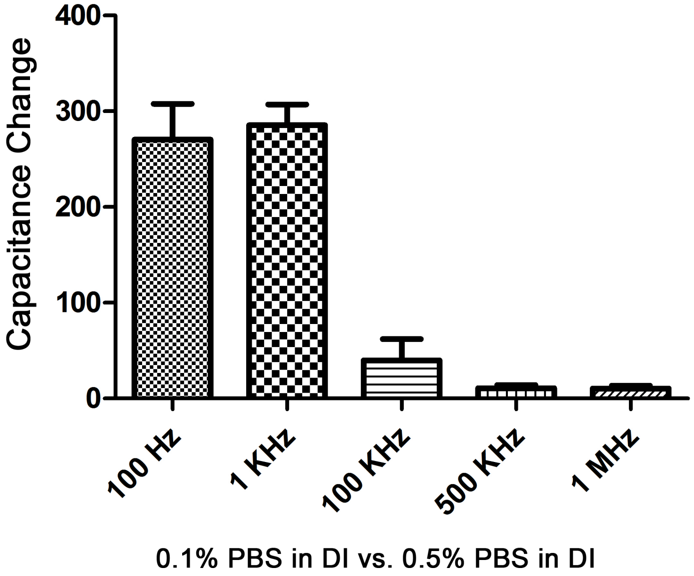

Figure S2. Capacitance spectrum. Capacitance was measured at various frequencies for 0.1% PBS (v/v in DI water) and 0.5% PBS (v/v in DI water) utilizing the microchips. The percentage change between 0.1% and 0.5% PBS for each frequency was calculated. 1 KHz showed the highest percentage of change with minimal noise. Error bars represent SEM (n=3).

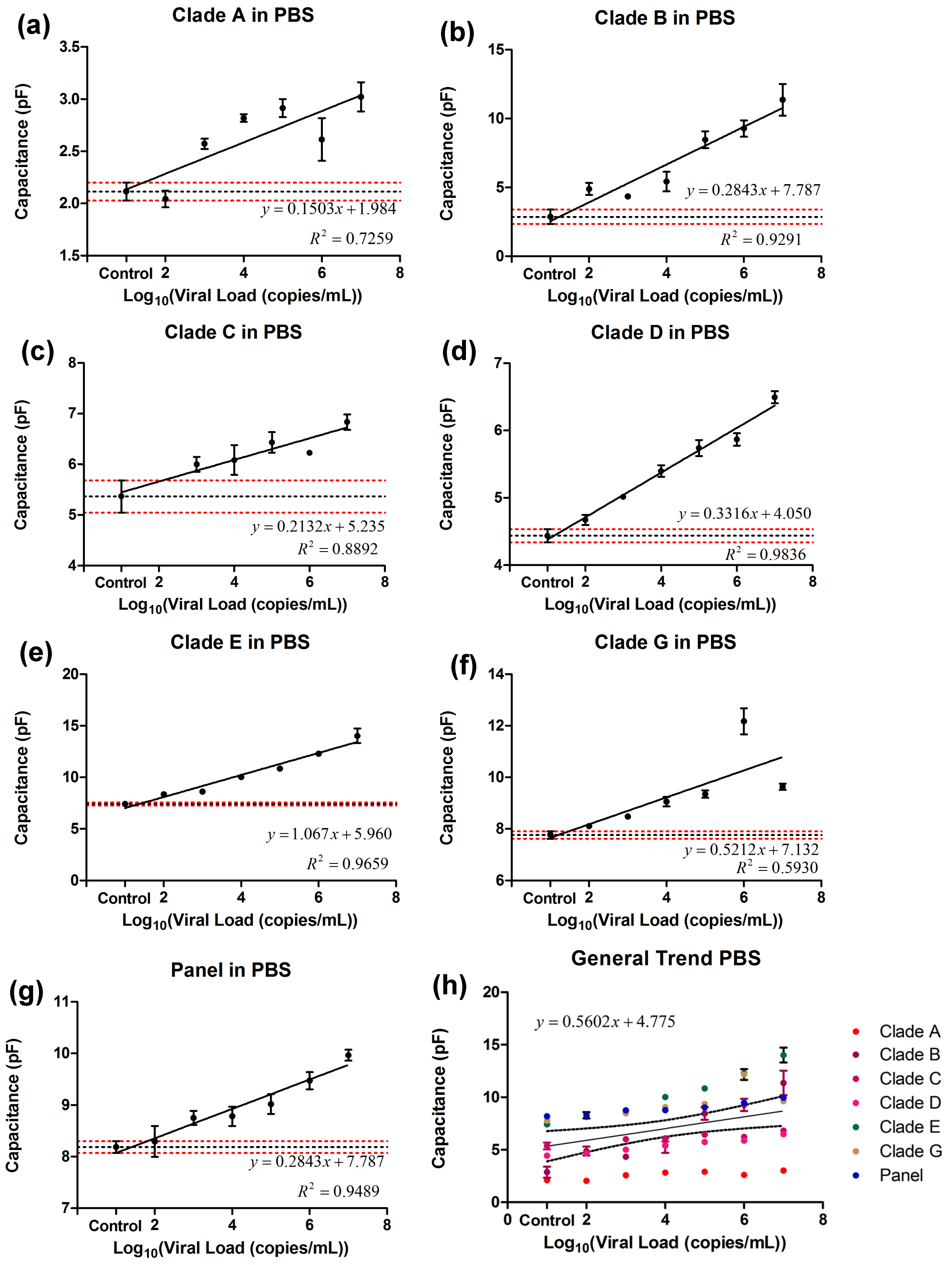

Figure S3. Microchip evaluation using HIV-1 subtypes spiked in PBS. Linear correlation between viral load of the samples and on chip capacitance measurements at 2V, 1 KHz for the various HIV-1 subtypes suspended in PBS was observed to possess coefficient of determinations between 0.5930 and 0.9825. The lowest dilutions which were statistically

significant from control in HIV-spiked PBS samples were 10^3 copies/ml (P=0.0005, n=8), 10^2 copies/ml (P=0.0104, n=8), 10^3 copies/ml (P=0.0007, n=8), 10^3 copies/ml (P=0.0002, n=8), 10^2 copies/ml (P=0.007, n=8), 10^3 copies/ml (P=0.002, n=8), and 10^3 copies/ml (P=0.010, n=8) for subtypes A **(a)**, B **(b)**, C **(c)**, D **(d)**, E **(e)**, G **(f),** and panel **(g)**, respectively. The Mann-Whitney method of analysis was used for statistical analysis **(h)** The general trend of all subtypes of HIV-1 spiked in plasma was obtained by generating a simple linear regression using all HIV-1 subtypes results. Error bars represent standard error of mean. Control samples are HIV-free PBS samples. Horizontal black and red dotted lines represent the average capacitance magnitude of the control samples and control ± standard error, respectively.

Table S1. Repeatability values of all samples. Repeatability of the measured capacitance magnitude for HIV-1 subtypes A, B, C, D, E, G and panel of HIV-1 subtypes (A, B, C, D, and circulating recombinant forms, CRF01_AE and CRF02_AG) suspended in PBS and plasma along with repeatability of both KSHV and EBV spiked in PBS and saliva.

**SAMPLE**	**Control**	**10^2**	**10^3**	**10^4**	**10^5**	**10^6**	**10^7**
**HIV Plasma**							
Clade A	99.53415	99.62101	98.56331	98.47051	97.60463	89.49217	97.91796
Clade B	97.86704	98.40532	90.17623	98.28948	96.1633	97.8002	96.0024
Clade C	98.83037	98.3913	98.19945	94.72765	97.10478	96.5466	92.99282
Clade D	98.91668	98.71153	98.56706	99.36258	99.13963	98.77071	97.57336
Clade E	98.19165	97.52271	99.51498	99.38408	99.32146	98.26951	98.25286
Clade G	99.46653	99.20168	98.39543	96.48991	98.04878	94.1654	94.9797
Panel	99.83852	99.36425	97.56944	98.74452	98.91458	99.14742	96.78587
**HIV PBS**							
Clade A	96.09309	96.28987	98.03735	98.74692	97.13949	92.71218	95.59963
Clade B	84.53736	91.8686	96.15257	88.46135	93.28543	94.0326	90.72033
Clade C	94.50651	93.86645	94.04818	93.2199	93.73872	93.22564	92.47898
Clade D	97.83783	98.4208	99.25657	98.4625	97.99136	98.49357	98.64713
Clade E	97.75643	97.3641	99.15968	98.07277	98.91052	99.128	95.15277
Clade G	98.19216	98.6915	98.93141	97.97962	98.51796	96.01048	98.77417
Panel	98.64314	96.52829	98.45988	97.91964	97.90149	98.28563	98.95642
**EBV**							
PBS	98.1945	99.34636	98.6045	99.08679	98.7844	98.29747	98.85982
Saliva	98.8647	98.06823	98.12311	99.00585	99.44802	99.68888	98.64504
**KSHV**							
PBS	98.39648	98.82138	97.76324	98.96626	98.70806	N/A	N/A
Saliva	98.59371	98.98451	98.85252	97.59333	98.80175	98.08022	N/A

The repeatability of the biological samples tested on our electrical sensing platform is defined as the percentage of variation in measured magnitudes of capacitance for each concentration of sample. It is given by,

$$\%\text{Repeatablity} = \frac{Mean\ of\ measured\ Capacitance}{Mean\ of\ measured\ capacitance + SEM} \times 100$$

The repeatability values ranged between 84.5-99.8%.

```
                  10         20         30         40         50         60         70         80         90
100
            ....|....| ....|....| ....|....| ....|....| ....|....| ....|....| ....|....| ....|....| ....|....|
....|....|
EU541617    MRVK~~~EKY QHLWRWGWRW GTMLLGMLMI CSATEKLWVT VYYGVPVWKE ATTTLFCASD AKAYDTEVHN VWATHACVPT DPNPQEVVLV
NVTENFNMWK
A           ~~~~~~~~~~ ~~~~~~~~~~ ~X.I...II. .NTA.N.... ......X.XD .XX....... .......... .......... ..X...XXME
...XE.....
B           ....GTRKN. ...~~~W.K. .M........ ...V.QT... .......... .N........ ....N..... .......... .......E.E
..........
D           ...RETKRN. ...~~~~.K. .......... ..V.G.S... .......... .......... ....KA.A.. I......... ......IK.E
..........
CM235 (E)   ....ETQMNW PN.~~~~.K. ..LI..LVI. ...SDN.... ........RD .D........ ...HE..... .......... ......IH.E
..........
G3          ....GTQRNW ...~~~~.T. W.LI..LVI. ...SNN.... ........ED .D.P...... ....S..R.. .......... ......IT.E
....T.....

                 110        120        130        140        150        160        170        180        190
200
            ....|....| ....|....| ....|....| ....|....| ....|....| ....|....| ....|....| ....|....| ....|....|
....|....|
EU541617    NDMVEQMHED IISLWDQSLK PCVKLTPLCV SLKCTD~~~~ ~~LKNDTNTN SSSGRMIMEK ~~GEIKNCSF NISTSIRGKV QKEYAFFYKL
DIIPID~~ND
A           .N..X...T. .........X ...Q...... T.D.SYN~~~ ~~IT.NIT~~ ~..ITNSXVN MRE......X .XT.EL.D.X R.X.SL....
.VVQ.XXNSG
B           .N..D..... .......... .......... T.S...K~~~ ~~VG...S.. ~N.RWDK... ~~........ ..T.NM.X.M ..Q..L....
.VV..E~~EG
D           .N........ .......... ......X... T.N...W~~~ ~~VT.T...~ ~~~~~~~~~~ ~~~~MT.... ..T.E..D.K KQVQ.L....
.VVK.N~~DT
CM235 (E)   .N.....Q.. V......... .......... T.H..NAN~~ ~~.T.VN.~~ ITNVPN.IGN ITD.VR.... .MT.EL.D.K ..VH.L....
..VQ.E~~DN
G3          .N........ ......E... .......... T.N..NVNCN SNVTSTG.SX GTNATCNI.E ~ANNL..... ..T.E..N.K KT...L..R.
.VV...~~GN

                 210        220        230        240        250        260        270        280        290
300
            ....|....| ....|....| ....|....| ....|....| ....|....| ....|....| ....|....| ....|....| ....|....|
....|....|
EU541617    ~~~~~~TTSY TLTSCNTSVI TQACPKVSFE PIPIHYCAPA GFAILKCNNK TFNGTGPCTN VSTVQCTHGI RPVVSTQLLL NGSLAEEEVV
IRSVNFTDNA
A           XNS~~~SSL. R.IN....AL .......T.. .......... .Y.....D. E.....L.K. .......... .......... ......GK.M
...E.I.N.V
B           KNNNSTF.D. R.I....... .......T.. .......... ...L...KD. K.......K. .......... K......... ..........
...E..SN..
D           DSN~~~D... R.IN....A. ......IT.. .......... ........E. K.......K. .......... K......... ........II
...E.L.N..
CM235 (E)   KKS~~~XSE. R.IN...... K.....I..D .......T.. .Y....X.D. N.......K. ..S.....X. K......... ........II
...EDL.N..
G3          NNV~~~SNN. R.IN..V.T. K........D .......... .......RD. N.T...Q.K. ..S....... K......... ......G.I.
...E.L....

                 310        320        330        340        350        360        370        380        390
400
            ....|....| ....|....| ....|....| ....|....| ....|....| ....|....| ....|....| ....|....| ....|....|
....|....|
EU541617    KTIIVQLNTS VEINCTRPNN NTRKKIRIQR GPGRAFVTIG K~IGNMRQAH CNISRAKWNA TLKQIASKLR EQFGNNKTII FKQSSGGDPE
IVTHSFNCGG
A           .N......E. .T........ ...RSV..~~ ...QT.YAT. DI..DI.... ..V.GSQ..R A.H.VVXQ.. .YW~~.T.X. .XN..XX.X.
XT.X......
B           R......... ...K.I.... ....G.H.~~ ......Y.T. DI..DI.... ....KQD..D ......E... ....~....V .SK.......
..M.T...A.
D           .I......E. .P...I..Y. ...QST..~~ ...Q.LF.TK V~..DI.... ....G.G..K ..Q.V.E..G NLLN~QT... ..P.......
.T........
CM235 (E)   .....H..K. ........S. X..TS...~~ ...Q..YRT. DI..DI.K.Y .E.NGT...E X...VTX..K .H.N~..... .QPP....L.
.TM.H...R.
G3          .V.....KT IG........ ....S...~~ ...Q..YAT. EI..DI.... ..V.GQE.QE M.QKVQAQ.E QV.~~..S.T .NS.X...L.
.T......R.

                 410        420        430        440        450        460        470        480        490
500
            ....|....| ....|....| ....|....| ....|....| ....|....| ....|....| ....|....| ....|....| ....|....|
....|....|
EU541617    EFFYCNSTQL FNSTWF~NST WSTEGSNNTE GSDTITLPCR IKQFINMWQE VGKAMYAPPI SGQIRCSSNI TGLLLTRDGG NN~~~NNGSE
IFRPGGGDMR
A           ...X..TSX. ....XVNGX~ ~TSSM..G.~ ~~~~XX.XX. .X.IX...XR ..Q.X.X..X Q.V.X.E... ...I...... X.~~~SXDX.
T.........
B           ......T.E. .....YA.G. D....G.K.N ~~~~.I.... .......... .......... .......... .......... KGNQXD.QT.
....V....K
D           ......T.R. .....KR.~~ ~NS.WXSDNT PDE....Q.. ...I...... .......... E.F.N..... .......... AIN~~SSQN.
T.........
```

```
CM235 (E)   ......T.R. ..N.CIX.E~ ~TMX.C.G.~ ~~~~.I...K ...I.....G A.Q....... X.R.N.V... ..I....... AI~~~.TTN.
T......NIK
G3          .....XTSG. ..ESG~~~~~ ~~~~.NDT.~ ~~~~.....K ...IVR...R ..Q....... A.D.T.R... .......... V.~~~.T.N.
T.........

                     510        520        530        540        550        560        570        580        590
600
            ....|....| ....|....| ....|....| ....|....| ....|....| ....|....| ....|....| ....|....| ....|....|
....|....|
EU541617    DNWRSELYKY KVVKIEPLGV APTKAKRRVV QREKRAVGIG ALFLGFLGAA GSTMGAASMT LTVQARQLLS GIVQQQNNLL RAIEAQQHLL
QLTVWGIKQL
A           .......... X......... .....R.... E.....XL. .V.I....T. .........I. ......K.X. ......S... ..........
K.........
B           N......... ...R.....I ...R...... .......... .......... .......... ......L... .......... ..........
..........
D           N......... ..X.L..I.L ...A...... E.....I.L. .........T. ......V.L. .......V.. .......... ..........
..........
CM235 (E)   .......... ...Q.....I ...R...... E......... .MIF...... .....X..I. .......... ......S... .....X....
..........
G3          .......... .I...K...I .....R.... E.G.....L. .V........ ....X..XI. ....V..... ......S... ....X.....
..........

                     610        620        630        640        650        660        670        680        690
700
            ....|....| ....|....| ....|....| ....|....| ....|....| ....|....| ....|....| ....|....| ....|....|
....|....|
EU541617    QARILAVERY LKDQQLLGIW GCSGKLICTT AVPWNASWSN KSLEQIWNHT TWMEWDREIN NYTSLIHSLI EESQNQQEKN EQELLELDKW
ASLWNWFNIT
A           ..XV...... .R........ ........P. N....S.... ...DE..DNM ..LQ..K..S ...IK.YE.. ....I...R. .KD.......
.......D.S
B           ...V...... .R........ .......... .....V..N. R.VDD..ENM ...Q.....S ......YT.. .......... .....A....
.N........
D           ........S. .......... .....H.... N....S.... R.VDE...NM .....E...D ...E.VY..L .V..I..... .....K..T.
.......S..
CM235 (E)   ...V...... ....KF..L. .....I.... .....ST... R.Y.E...NM ..I..E...S ...NQ.YEIL T...D..DR. .KD.......
.......D..
G3          ...V...... .......... .......... N....T.... ..Y.E..DNM ..IQ.E..VS ...QQ.Y... .......... ..D..A....
.......D..

                     710        720        730        740        750        760        770        780        790
800
            ....|....| ....|....| ....|....| ....|....| ....|....| ....|....| ....|....| ....|....| ....|....|
....|....|
EU541617    NWLWYIKLFI MIVGGLVGLR IVFAVLSVVN RVRQGYSPLS FQTHLPIPRG PDRPEGIEEE GGERDRDRSI RLVNGSLALI WDDLRSLCLF
SYHRLRDLLL
A           K......I.. ......I... .........I. .......... ....T.N... L...GR..X. ...Q..G... ...S.F...A .....N....
.......FI.
B           E......I.. .......... ........I.. .......... ......AQ.. ....G..... ...S.....G .....F..I. .V........
..........
D           Q......I.. .....XI... .......... .......... ...L..A..E .......... ......G... .....LS... .....N....
...X....I.
CM235 (E)   .......I.. ..I...I... .I.....IX. .......... ...PSHHQ.E .....R...G ...QG....V X..S.F.X.A ..........
.......FI.
G3          K......I.. ......I... ........I.. .......... ...LTHHQ.E .....R...G ...QX....V ...S.F...A .....N....
........V.

                     810        820        830        840        850        860        870        880
            ....|....| ....|....| ....|....| ....|....| ....|....| ....|....| ....|....| ....|....| ....|...
EU541617    IVTRIVELLG RR~~~~~~~G WEALKYWWNL LQYWSQELKN SAVSLLNATA IAVAEGTDRV IEVVQEAYRA IRHIPRRIGQ GLERILL*
A           .AA.T..... HSSLKGLRL. ..G...LG.. .X..GR...T ..IN.FDTI. .V..GW.... ...G.RLG.. .LN.....R. ....A..*
B           .......I.. ..~~~~~~~. ..I....... ....I..... .........I. ...G.....I ..AFRSIF.. .L...T..R. ....S..*
D           .A........ ..~~~~~~~. ...I..L... ....I..... .....F.TI. .........A ..L..R.X.. .LN..V..R. ....A..*
CM235 (E)   .AA.T..... .XSLKGLRR. ..G...LG.X .L..G....I ..I...X.A. .T..GW.... ...A.G.W.. .L......R. .F..A..*
G3          .AA.TA...R .SSLQGLRL. ..G...L... .L..GR.... ..IN.IDTI. ....NW.... ...A.G.C.. .LN.....R. ....A..*
```

Figure S4. Sequence comparisons. Protein translation of gp120 nucleotide sequences of HIV-1 subtypes A, B, D, E and G, as compared to the translation of gp120 from EU541617, the virus that was used to generate ab53937, the goat polyclonal antibody to gp120 used in the experiments. Population sequencing was performed and protein sequences were translated from nucleotide sequences using BioEdit. Conserved proteins between viruses are denoted by

dots. Where there is a difference in protein at a specific position, the single letter code of the amino acid that is present is instead listed. “X” signifies that the identity of the protein at the position is unknown, due to the presence of an ambiguous nucleotide in sequence from which the translation was generated.

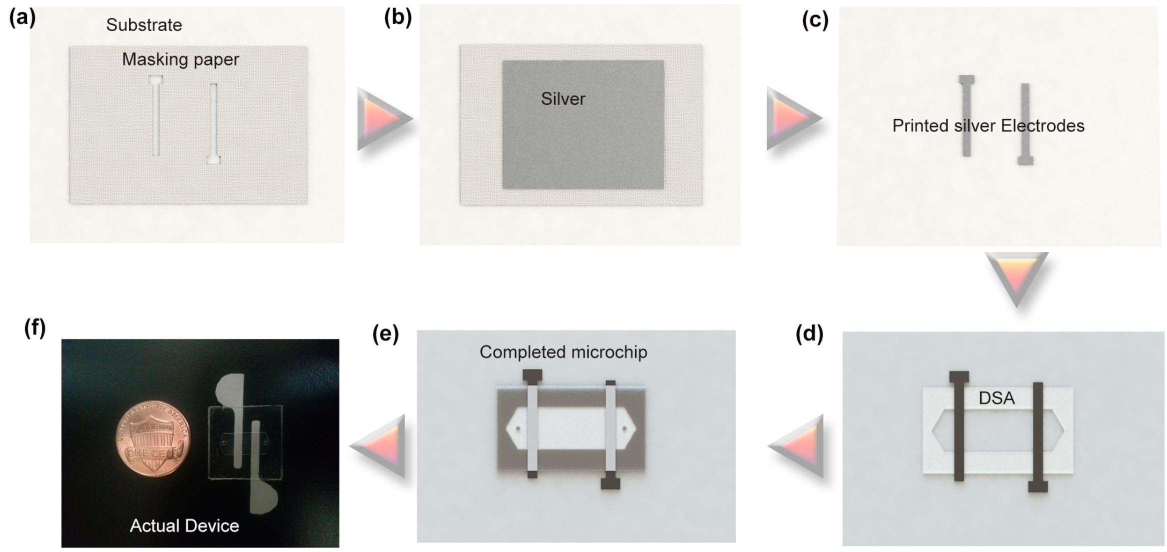

Figure S5. Screen printed flexible plastic microchip fabrication. **(a)** Electrode designs are patterned on a masking paper using a laser cutter. The patterned masking paper is firmly fixed over a flexible transparency sheet. **(b)** Silver paste is applied over the mask lavishly and is evened out. **(c)** The mask is gently removed from the substrate and the electrode containing substrate is baked in an oven for 3 hours at 175° C. **(d)** Patterned double sided adhesives (DSA) are used to form the channels of the microchip. One side of the adhesive is attached to the printed electrode substrate, while the other **(e)** is attached to another substrate, which contains openings for the inlet and outlet of the chip. **(f)** An actual microchip placed against a US one cent coin is shown. The device thickness is less than the coin thickness.

Table S2. Patient sample results obtained using the microchip and RT-qPCR. Quantitative viral load measurement for the patient samples with both RT-qPCR and the printed flexible plastic microchip are shown in this table. Microchip results are mean ± standard error of mean value.

Patient no.	Microchip result	RT-qPCR
**Patient 1**	$(1.9 \pm 0.40) \times 10^3$	3.3×10^4
**Patient 2**	Not Detectable	Not detectable
**Patient 3**	$(1.07 \pm 0.19) \times 10^3$	4.025×10^3
**Patient 4**	$(7.37 \pm 0.2) \times 10^3$	1.5×10^4
**Patient 5**	$(2.13 \pm 0.35) \times 10^5$	1.5×10^5
